# Large-scale Isolation of Exosomes Derived from NK Cells for Anti-tumor Therapy

**DOI:** 10.21769/BioProtoc.4693

**Published:** 2023-06-05

**Authors:** Heyong Luo, Jing Zhang, Anqing Yang, Weiwei Ouyang, Shiqi Long, Xiaojin Lin, Na Yang, Zhiru Yang, Yingchun Zhang, Wei Yang, Qiyuan Che, Yuxin Yang, Ting Guo, Xing Zhao

**Affiliations:** 1 Center for Tissue Engineering and Stem Cell Research, Guizhou Medical University, Guiyang, China; 2 Department of Immunology, College of Basic Medical Sciences, Guizhou Medical University, Guiyang, China; 3 Department of Biology, College of Basic Medical Sciences, Guizhou Medical University, Guiyang, China; 4 Department of Thoracic Oncology, The Affiliated Hospital/The Affiliated Cancer Hospital of Guizhou Medical University, Guiyang, China; 5 Department of Oncology, Guizhou Medical University, Guiyang, China; 6 Development and Related Diseases of Women and Children Key Laboratory of Sichuan Province

**Keywords:** Exosome, Natural killer cells, Tangential flow filtration, Ultracentrifugation, Immunotherapy

## Abstract

Exosomes are lipid bilayer–enclosed vesicles, actively secreted by cells, containing proteins, lipids, nucleic acids, and other substances with multiple biological functions after entering target cells. Exosomes derived from NK cells have been shown to have certain anti-tumor effects and potential applications as chemotherapy drug carriers. These developments have resulted in high demand for exosomes. Although there has been large-scale industrial preparation of exosomes, they are only for generally engineered cells such as HEK 293T. The large-scale preparation of specific cellular exosomes is still a major problem in laboratory studies. Therefore, in this study, we used tangential flow filtration (TFF) to concentrate the culture supernatants isolated from NK cells and isolated NK cell–derived exosomes (NK-Exo) by ultracentrifugation. Through a series of characterization and functional verification of NK-Exo, the characterization, phenotype, and anti-tumor activity of NK-Exo were verified. Our study provides a considerably time- and labor-saving protocol for the isolation of NK-Exo.

## Background

Adoptive NK cell therapy is a promising new approach for the treatment of hematological and solid malignancies, whose safety and efficacy have been tested in multiple studies mainly by utilizing autologous or allogeneic NK cells (van Vliet et al., 2021; Laskowski et al., 2022). Although the efficacy of adoptive NK cell therapy in the treatment of hematologic tumors has been verified, there are limitations to their application in solid tumors (van Vliet et al., 2021). Due to the existence of various biological barriers in the body, the migration and infiltration of NK cells to tumors are insufficient (Kang et al., 2021; Russo et al., 2021). The tumor microenvironment (TME) has immunosuppressive effects on NK cell function (Terrén et al., 2019; Di Pace et al., 2020); in addition, storing and transportation of NK cells is difficult, which increases the cost of treatment.

Exosomes are vesicles with a phospholipid bilayer membrane structure that are actively secreted by a variety of cells and carry the characteristic substances of their source cells (Shoae-Hassani et al., 2017; Dai et al., 2022; Rezaie et al., 2022). NK cell–derived exosomes (NK-Exo) have some significant advantages over NK cell therapy. Due to their nanoscale size and good tissue permeability, NK-Exo can cross some biological barriers such as the blood-tumor and blood-brain barriers (Yang et al., 2021). These are difficult for NK cells to cross (Nayyar et al., 2019; Yong et al., 2019; Kang et al., 2021), leading to the possibility of NK-Exo having a better tumor-targeting effect. It has been demonstrated that NK-Exo express perforin, granzyme B, Fas/FasL, and other anti-tumor substances (Zhu et al., 2017; Di Pace et al., 2020; Federici et al., 2020; Han et al., 2020). NK-Exo is not restricted to the TME and retains its original anti-tumor activity (Federici et al., 2020). In addition, NK-Exo has simpler preservation conditions than NK cells. The potential of implementing exosomes as an anti-tumor therapy has been demonstrated in several clinical trials (Chen et al., 2020; Nikfarjam et al., 2020; Lee et al., 2021; Rezaie et al., 2022). Therefore, we speculate that NK-Exo could also be used as a *cell-free* therapy to provide alternative anti-tumor immunotherapy.

However, the dose-demand for NK-Exo when used in anti-tumor therapy is enormous (Kibria et al., 2018; Kimiz-Gebologlu and Oncel, 2022). At present, laboratory methods for exosome isolation include ultracentrifugation, size exclusion chromatography, and exosome acquisition kits. However, it is challenging to isolate enough exosomes in supernatants in laboratory studies, such as exosomes derived from NK cells. Therefore, the large-scale preparation of exosomes is a key issue to be investigated urgently for further application.

Our protocol focuses on the following components: first, NK cell supernatants are collected and concentrated by tangential flow filtration (TFF) system, allowing us to obtain NK-Exo by ultracentrifugation. The interception efficiency of TFF system is evaluated by nanoflow assay. The obtained NK-Exo is then characterized to evaluate their phenotype, as well as morphological and structural integrity. Finally, the anti-tumor functionality of NK-Exo is evaluated in vitro. This protocol provides an efficient and convenient method to concentrate and isolate NK-Exo from large-scale NK cell culture supernatants, which can provide enough NK-Exo for solid tumor therapy.

## Materials and reagents

1. 0.22 μm filter (PALL, catalog number: 4612)
2. 50 mL centrifuge tube (NEST, catalog number: 602001)
3. 0.45 μm PVDF (Millipore)
4. RPMI 1640 medium (Gibco)
5. Fetal bovine serum (FBS) (Gibco)
6. 1% penicillin/streptomycin (Gibco)
7. Centrifuge ware (Hitachi, catalog number: 3 29607A)
8. Annexin -FITC/PI Apoptosis Detection Kit (Absin, catalog number: abs50001)
9. Cell Counting kit 8 (Dojindo, catalog number: CK04)
10. FITC-conjugated mouse anti-human CD107a (BioLegend, catalog number: 328606)
11. PBS (meilunbio, catalog number: MA0015)
12. PE-conjugated mouse anti-human CD56 (BioLegend, catalog number: 304605)
13. PKH67 green fluorescent cell linker Midi kit (Sigma-Aldrich, catalog number: MIDI67)
14. Silica nanosphere cocktail (NanoFCM, catalog number: S16M-Exo)
15. DAPI (Sigma, catalog number: D9592)
16. 4% paraformaldehyde (ChemCruz, catalog number: sc281692)
17. 12% SDS-PAGE (TGX Stain-Free FastCast Acrylamide Kit, 12%) (Bio-Rad, catalog number: 161-0185)
18. SDS-PAGE sample loading buffer (5×) (Beyotime, catalog number: P0015L)
19. SDS-PAGE running buffer powder (Servicebio, catalog number: G2081-1L)
20. SDS-PAGE transfer buffer powder (Servicebio, catalog number: G2017)
21. Western blocking buffer (Beyotime, catalog number: P0023B-100ml)
22. Uranyl acetate (HSA Biotech)
23. RIPA (Solarbio)
24. PMSF (Sigma)
25. TEMED (Bio-Rad)
26. TBST (20×) (Solarbio)
27. Loading buffer (5×) (Beyotime)
28. 10–180 kD marker (Absin)
29. Western chemiluminescent HRP substrate (Millipore, catalog number: WBKIS0100)
30. SKOV3 cells (China Center for Type Culture Collection, CCTCC)
31. OV-90 cells (China Center for Type Culture Collection, CCTCC)
32. Primary and secondary antibodies (Table 1)
  **Table 1.** Primary and secondary antibodies

Antibody	Species	Host	Company	Catalog number	Dilution ratio
CD63	Human	Rabbit	Abcam	ab134045	1:1,000
CD81	Human	Rabbit	CST	56039s	1:1,000
Calnexin	Human	Rabbit	Biodragon	BD-PT0613	1:1,000
CD56	Human	Rabbit	CST	99746s	1:1,000
Perforin	Human	Rabbit	Biodragon	BD-PT5792	1:1,000
Rabbit-HRP	Rabbit	Goat	Absin	Abs20040	1:5,000

## Equipment

1. Transmission electron microscope (Hitachi, catalog number: HT-7700)
2. Ultracentrifuge (Hitachi, catalog number: CPN100N)
3. ChemiDoc XRS high sensitivity chemiluminescence instrument (Bio-Rad Laboratories)
4. Filter cassettes (100 kD) (Sartorius, catalog number: VF05H4)
5. Flow cytometer (Beckman Coulter Inc., model: FC500)
6. Fluorescence microscope (Olympus BX51)
7. Nanoflow cytometry (NanoFCM)
8. P28S rotor (Hitachi)
9. Peristaltic pump (Millipore, model: XX80EL005）
10. Autoclave (MEDNIF, model: LS-B50L)
11. Ultraviolet irradiation (SJMAEA)
12. Centrifuge (Eppendorf)
13. Biosafety cabinet (Thermo Scientific)
14. PowerPac^TM^ (Bio-Rad)

## Software

1. Fiji: ImageJ 1.53a
2. GraphPad Prism 8
3. NanoFCM software (NanoFCM Profession V1.0)
4. FlowJo 10

## Procedure

- **NK cell culture supernatants preparation**
  1. Maintain the NK cells expanded in vitro in RPMI 1640 medium supplemented with 5% FBS and 1% penicillin/streptomycin.
  2. Change the FBS to exosome-free FBS on the 14th day of culture and continue the culture for two days.
*Note: Exosome-free FBS is obtained by centrifugation of FBS at 100,000× g for 16 h followed by the collection of supernatants.*
  3. Adjust cell density to 1 × 10^9^ cells/500 mL and collect the cell culture supernatant for pretreatment.
  4. Centrifuge the cell culture supernatant at 300× *g* for 10 min to remove cells and then transfer the supernatant to a new centrifuge tube.
*Note: The centrifugal force of the first step should be controlled below 350× g to avoid damage to NK cells.*
  5. Centrifuge the supernatant obtained in the previous step again at 2,000× *g* for 10 min to remove cell debris, and then transfer the supernatant to the sample cup of the TFF system for subsequent treatment.
*Note: The volume of supernatant can be increased by increasing the number of sample cups or by adding liquid multiple times to a single sample cup.*
- **Concentration of NK cell culture supernatants by TFF system**
*Note: All the following steps are performed at 4 °C; for reference, we performed all steps in cold storage.*
  1. Connect the sample cup, the liquid inlet pipe, the peristaltic pump, the filter cassettes (100 kD), the liquid outlet pipe, the waste liquid pipe, and the waste liquid cup successively, and install the pressure indicator at the connection of the liquid outlet pipe to complete assembly of the TFF system (Kim et al., 2021).
*Note: The sample cup should be autoclaved in advance, and the entire TFF system should be sterilized by ultraviolet irradiation 30 min before operation.*
  2. Add the pretreated cell culture supernatants from steps A4–A5 into the sample cup, start the peristaltic pump, and concentrate at a pressure of 2.5 bar until the concentrated volume is approximately 13 mL.
  3. Transfer the concentrate to a 50 mL centrifuge tube and add 10 mL of PBS into the sample cup.
  4. Reduce the pressure of the peristaltic pump to 1 bar.
  5. After recirculating the TFF system approximately five times, shut down the peristaltic pump and transfer PBS to the above 50 mL centrifuge tube.
*Note: All residual fluid in the pipeline should be drained as far as possible to improve exosome recovery.*
- **Preparation of NK-Exo using ultracentrifugation**
*Note: All following steps should be performed at 4 °C as much as possible; steps in the biosafety cabinet should be performed on ice.*
  1. Transfer the concentrated solution from step B5 to an ultra-speed centrifuge–fitted centrifuge tube and centrifuge at 10,000× *g* for 30 min at 4 °C; then, filter through a 0.22 μm filter.
*Note: The centrifuge tube should be irradiated by UV 30 min in advance, and all following steps should be performed aseptically in the biosafety cabinet.*
  2. Centrifuge the liquid from the previous step at 100,000× *g* for 70 min at 4 °C.
  3. Remove the supernatants, re-suspend the exosome pellet with 20 mL of precooled PBS, and centrifuge again at 100,000× *g* for 70 min at 4 °C.
*Note: The suspended liquid should account for at least 1/2 of the centrifugal tube volume, so as not to damage the centrifugal tube due to high centrifugal force.*
  4. Remove and re-suspend the supernatants with 500 μL of PBS. Then, immediately use or store at -80 °C.
*Note: NK-Exo should be used as soon as possible or stored at -80 °C for up to three months.*
- **Detection of particle concentration, particle size, and phenotypes by nanoflow cytometry**
  1. Calibrate the particle concentration of the instrument with 200 nm PE and AF488 fluorescent-conjugated polystyrene beads and calibrate the particle size of the instrument with silica nanosphere cocktail.
  2. Dilute NK-Exo serially to an optimal recording range of 2,000–12,000 particles/min during the final detection and record all particles passing through the 1 min interval during each detection.
*Notes:*
    - *NK-Exo should be diluted at a ratio of 1:10, 1:100, and 1:1,000 until its detection rate reaches 2,000–12,000 particles/min.*
    - *Incubation should be done in dark conditions.*
  3. Then, wash the dyeing mixture with PBS and centrifuge at 100,000× *g* for 70 min at 4 °C.
*Note: PBS should account for at least 1/2 of the centrifugal tube volume, so as not to damage the centrifugal tube due to high centrifugal force.*
  4. Re-suspend NK-Exo with 50 μL of PBS and test by nanoflow cytometry.
  5. Using a standard curve, convert the flow rate and lateral scattering intensity to the corresponding concentrations and size in NanoFCM software.
- **NK-Exo detection by transmission electron microscopy**
  1. Re-suspend exosomes (> 10^9^/mL) purified from NK cell supernatants in 50 μL of PBS.
  2. Obtain a 10 μL sample and drop it on a copper grid. After 1 min of precipitation, use a filter paper to remove the excess liquid.
  3. Obtain 10 μL of uranyl acetate and add it onto the copper grid to precipitate for 1 min; use a filter paper to remove the excess liquid.
  4. After drying at room temperature for several minutes, the image results are obtained using a transmission electron microscope (100 kV).
- **NK-Exo detection by western blot analysis**
  1. Add 150 μL of RIPA containing 1.5 μL protease inhibitor (PMSF) to NK-Exo. After leaving to crack on ice for 5 min, collect the supernatants after centrifugation at 13,000× *g* for 20 min.
*Note: These steps are performed at 4 °C.*
  2. Add 5× loading buffer to the supernatants obtained in the previous step and heat at 95 °C for 10 min.
  3. Separate the products from the previous step by 12% SDS-PAGE and use 10–180 kD marker as an indicator ruler.
*Note: Choose the gel percentage according to the molecular weight of the protein of interest.*
  4. Run the gel in running buffer at 80 V for approximately 30 min, then adjust the voltage to 120 V and run for 50 min until the loading dye reaches the bottom of the gel.
*Note: The run time may vary according to the equipment used or type and percentage of gel.*
  5. Transfer the gel using a PVDF membrane at 300 mA for 80 min in transfer buffer.
  6. Block the membrane with Western blocking buffer at room temperature for 2 h.
  7. Incubate the membranes with primary antibodies (Table 1) overnight and shake at 4 °C.
  8. Wash the membranes in 1× TBST three times for 10 min each time.
  9. Incubate the membrane with the appropriate secondary antibody (Table 1) and shake at room temperature for 2 h.
  10. Wash the membrane three times with 1× TBST for 10 min each time
  11. Images are displayed using Western chemiluminescent HRP substrate under chemiluminescence instrument.
  For phenotype analysis, NK-Exo is first suspended in 50 μL of PBS according to the concentration of 1 × 10^9^ particles/mL, mixed with 5 μL of PE-conjugated mouse anti-human CD56 (a marker of NK cells) and FITC-conjugated mouse anti-human CD107a (a marker for NK cell degranulation), and incubated at 37 °C for 30 min.
- **NK-Exo uptake**
  1. Stain NK-Exo with PKH67 green fluorescent cell linker Midi kit and mix NK-Exo with diluent buffer (Diluent C).
*Note: Do not use a vortex to mix vigorously; use a pipette to gently mix.*
  2. Add the NK-Exo mixture from the previous step into the pre-prepared 2× PKH67 dye solution, mix gently, and incubate for 4 min.
*Note: NK-Exo must be added to the dye solution—the order must not be changed—and the dye must be mixed from time to time during incubation.*
  3. Add an equal volume of FBS to stop staining for 1 min.
  4. Centrifuge at 100,000× *g* for 70 min to wash the residual dye.
*Note: If the dyed exosomes are not used immediately, they can be stored at -80 in the dark for one week.*
  5. Culture SKOV3 cells in 24-well plates with 5 × 10^4^ cells per well and add 20 μg of NK-Exo to each well for a 6 h co-culture at 37 .
  6. After incubation, wash the cells with PBS and fix in 4% paraformaldehyde, and stain the nuclei with DAPI.
*Note: Washing with PBS should be sufficient to avoid residual dye affecting the experimental results.*
  7. Image using a fluorescence microscope and quantify the uptake of NK-Exo by SKOV3 cells using ImageJ 1.53a software.
- **Functional characterization of NK-Exo**
  1. To evaluate the cytotoxicity of NK-Exo on tumor cells, add SKOV3 and OV-90 ovarian cells into 96-well plates at the concentration of 1 × 10^4^ cells per well.
  2. Add NK-Exo with a concentration of 80 μg/mL to each well and incubate at 37 °C for 24 h.
  3. Add 10 μL of Cell Counting kit 8 (CCK8) reagent to each well and incubate at 37 °C for 2 h.
*Note: For this suspension volume, 10 μL of CCK8 reagent was added to each well. For other volumes, a corresponding volume of CCK8 is added.*
  4. Measure cell viability at 450 nm. Repeat each experiment in triplicate and analyze statistically using GraphPad Prism 8 software.
  5. Calculate cell survival rate using the following formula:
    Survival rate = (OD_experiment_ - OD_blank_)/(OD_control_ - OD_blank_) × 100%
  6. Use an Annexin -FITC/PI Apoptosis Detection kit to detect the proportion of tumor cell apoptosis induced by NK-Exo.
  7. Pre-treat SKOV3 and A2780 ovarian cancer cells with 80 μg/mL of NK-Exo for 24 h.
  8. Wash cells twice with 1× PBS and re-suspend in 1× binding buffer.
  9. Stain with 5 μL of Annexin -FITC for 10 min in the dark, followed by 5 μL of PI for 5 min before flow cytometry analysis.
  10. Use a flow cytometer for quantification of apoptotic cells and analyze with FlowJo 10.

## Data analysis

- **Quantification of interception efficiency of TFF system**
  1. The particle size and concentration of the filtrate from the TFF system and the prepared NK-Exo were detected by nanoflow to evaluate the interception efficiency of exosomes in the TFF system.
  2. Record all particles passing through the 1 min interval during each detection (Table 2).
    **Table 2.** Comparison of particle distribution in ultrafiltered solution and concentrated solution after TFF system concentration. The particles in the filtrate are below 70 nm, while the particle size of NK-Exo ranges from 60 to 90 nm. By comparing the total particle numbers of the two, it can be concluded that the retention efficiency is 94.40%.

Concentrate		Ultrafiltrate	
**Size (nm)**	**Events**	**Size (nm)**	**Events**
40–50	0	40–50	3
50–60	577	50–60	177
60–70	1474	60–70	75
70–80	1205	70–80	17
80–90	661	80–90	5
90–100	397	90–100	1
100–150	592	100–150	3
150–250	86	150–250	1
Total	4992	Total	282
  3. Convert and analyze the collected data by NanoFCM software to obtain the particle concentration and particle size (Figure 1A–1B).
    **Figure 1.**
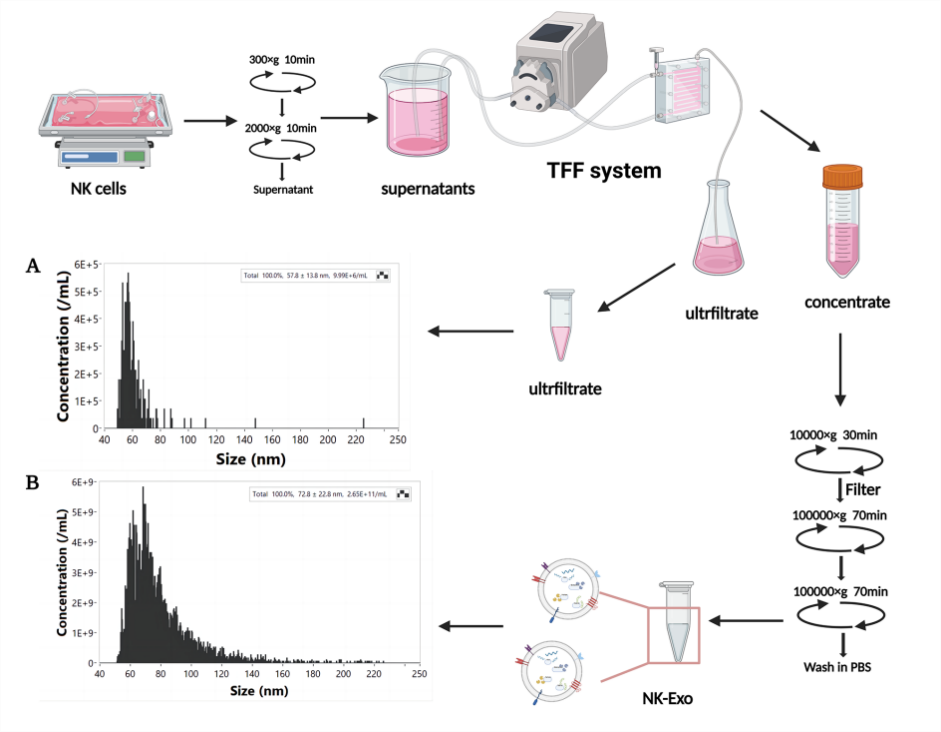
Isolation and nanoflow detection of exosomes derived from NK cells (NK-Exo). The particle concentration and size of ultrafiltrate (A) and NK-Exo (B) were detected by nanoflow.
- **Characterization of NK-Exo**
  1. Morphological images of exosomes were collected by transmission electron microscopy (Figure 2A). They show a typical *saucer shape* with a particle size of approximately 80 nm, which was consistent with nanoflow results. The expression of exosome surface markers CD63, CD81, and TSG101 were identified by western blot (Figure 2B).
    **Figure 2.**
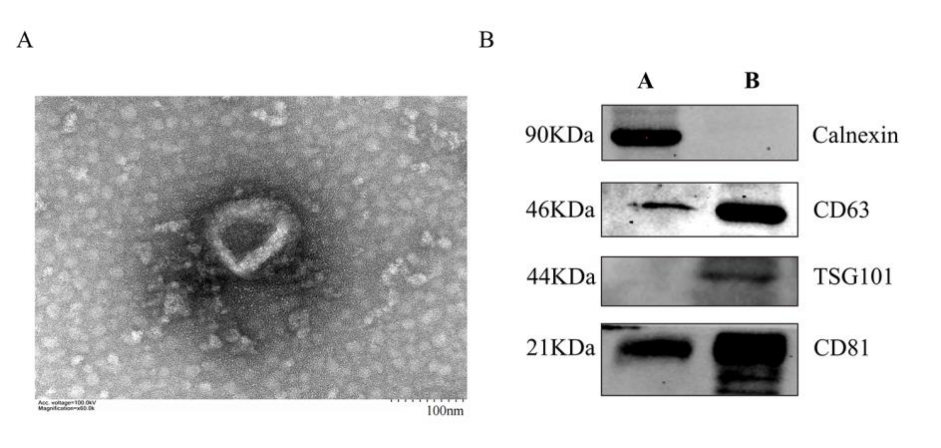
Characterization of NK cell–derived exosomes (NK-Exo). (A) Transmission electron microscopy images of NK-Exo. Scale bar: 100 nm. (B) Western blot analysis of CD81 (22 kDa), CD63 (46 kDa), TSG101 (44 kDa), and calnexin (90 kDa) expression on NK-Exo (A: cell lysate, B: NK-Exo).
- **Internalization of NK-Exo**
  1. Fix SKOV3 cells treated with PKH67-labeled NK-Exo for 6 h with 4% paraformaldehyde for 30 min and wash three times with PBS. Stain with DAPI at 0.1 μg/mL for 20 min and wash three times with PBS.
  2. Take immunofluorescent images of those labeled cells (Figure 3A).
  3. Quantify fluorescence intensity and area using ImageJ software to calculate uptake rate of PKH67-labeled NK-Exo (Figure 3B).
    **Figure 3.**
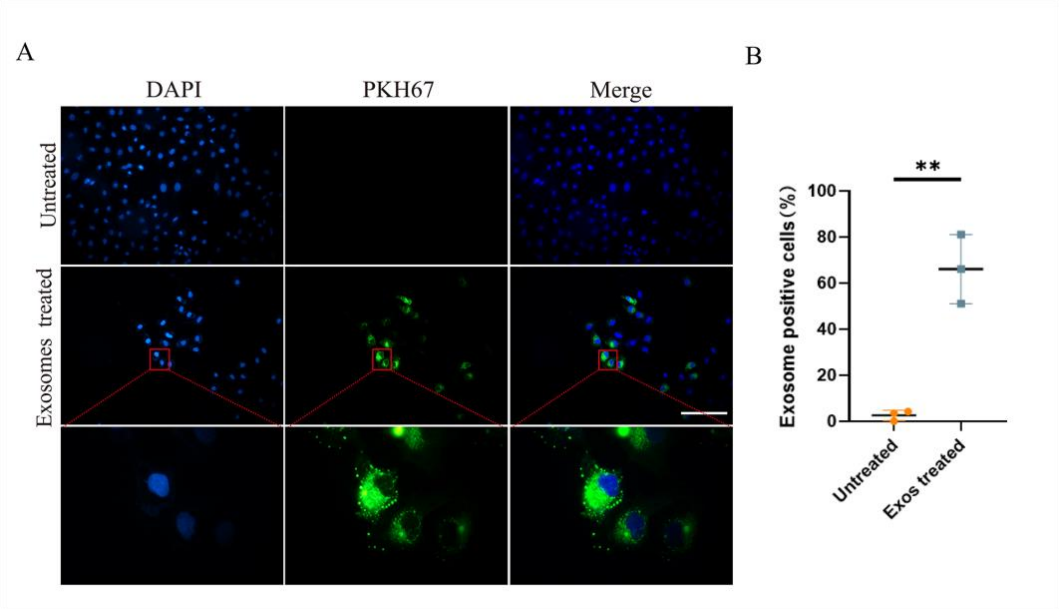
Cellular uptake of NK cell–derived exosomes (NK-Exo) by ovarian cancer cells. (A) NK-Exo uptake by SKOV3 cells. SKOV3 cells were stained blue by DAPI and NK-Exo were stained green by PKH67. (B) NK-Exo uptake quantified by green fluorescence intensity (10×, scale bar: 200 μm; 100×, scale bar: 40 μm, n = 3, mean ± SEM, t test, **p < 0.01). Quantification of fluorescence intensity demonstrated significant uptake of PKH67-labeled NK-Exo by SKOV3 cells and the uptake rate reached 50% in 6 h.
- **Functional verification of NK-Exo**
  1. Detect the expression of typical NK cell marker CD56 and degranulation marker CD107a on NK-Exo by nanoflow analysis (Figure 4A).
  2. Detect the expressions of CD56 and perforin in NK-Exo by western blot (Figure 4B). NK cells are known to exert their cytolytic effect through the release of effector molecules such as perforin, which may contribute to the cytotoxicity of NK-Exo against tumor cells.
  3. Detect the cytotoxicity of NK-Exo to SKOV3 and OV-90 ovarian cancer cells by CCK8 assay (Figure 4C).
  4. Use flow cytometry to detect NK-Exo–mediated apoptosis of SKOV3 and A2780 ovarian cancer cells (Figure 4D). Apoptosis analyzed by flow cytometry confirmed that NK-Exo can induce apoptosis in SKOV3 and A2780 cells; the proportion of apoptotic cells was comprised of both late apoptotic (upper right quadrant) and early apoptotic cells (bottom right quadrant).
    **Figure 4.**
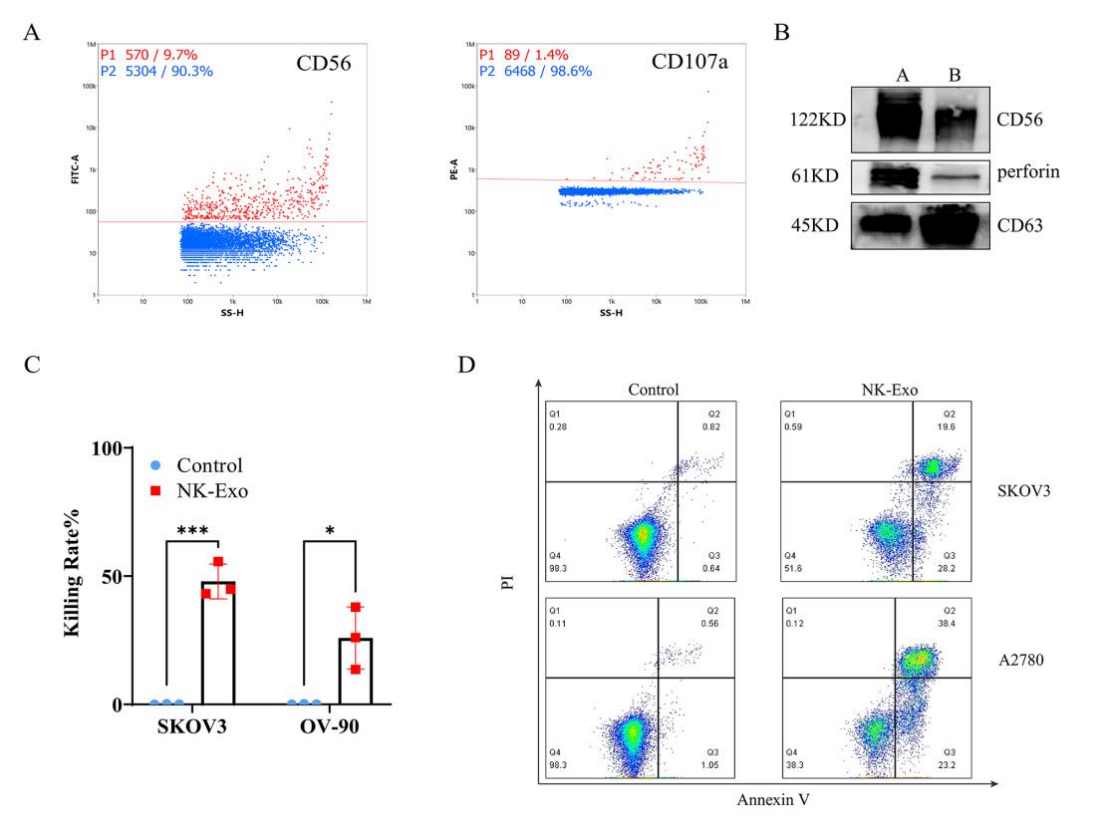
Functional characterization of NK cell–derived exosomes (NK-Exo). (A) Expression of CD56 and CD107a on the surface of NK-Exo was detected by nanoflow. Red dots represent positive signals. (B) Western blot analysis of CD56 (122 kD) and perforin (61 kD) expression (A = cell lysates, B = NK-Exo). (C) CCK-8 assay of NK-Exo against SKOV3 cells and OV-90 cells (n = 3, mean ± SEM, t-test, *p < 0.05, ***p < 0.001). (D) Apoptosis analysis of SKOV3 and A2780 cells after NK-Exo treatment by flow cytometry.

**Discussion**

The traditional exosome separation method is ultracentrifugation. However, this method has limitations on the volume for application, and can only achieve a large number of exosomes by using a small volume and multiple centrifuges, which is very costly and therefore poses a great challenge to the application of exosomes in clinical treatment (Liang et al., 2018).

To overcome these issues, we used a TFF system combined with a conventional ultra-high speed centrifugal method to concentrate a large volume of cell culture supernatants. Concentration ensures that exosomes are retained while smaller impurities and excess water are removed. This step can achieve 50–160-fold concentration of cell culture supernatants and adequately trap NK-Exo to ensure no exosome loss.

Before concentration, we performed a two-step low-speed centrifugation on cell culture supernatants to remove large cell fragments. This step reduces the wastage of the TFF system and speeds up the concentration. The inlet pressure was maintained at 2.5 bar for concentration to avoid damaging the structural integrity of NK-Exo. The pressure is then reduced at a slow rate for final concentration before completing enrichment to improve the recovery rate of NK-Exo. NK-Exo were then separated from the concentrated solution by ultracentrifugation. The particle concentration and size of tangential flow filtrate and NK-Exo were detected by nanoflow, and it was determined that the interception efficiency of TFF system for NK-Exo was higher than 90%.

As confirmed by the characterization results of NK-Exo, this method does not damage the structural integrity of NK-Exo, which still maintained their characteristic morphology and size, with removal of most of the cell debris contamination.

In this study, NK-Exo were further functionally validated. First, NK-Exo were confirmed to be able to enter ovarian cancer cells and further exert their effect. We detected some cytotoxic substance in NK-Exo and demonstrated NK-Exo were able to exert cytotoxic effects on OC cells and induce apoptosis in vitro. Therefore, NK-Exo isolated from large-scale cell culture supernatants by our protocol can maintain their complete morphological structure, phenotype, and anti-tumor activity, all of which provide a possibility for the application of NK-Exo in clinical tumor therapy.
